# *METTL3* Is Associated With the Malignancy of Esophageal Squamous Cell Carcinoma and Serves as a Potential Immunotherapy Biomarker

**DOI:** 10.3389/fonc.2022.824190

**Published:** 2022-04-27

**Authors:** Yubin Zhou, Songhe Guo, Yiqiu Li, Fangfang Chen, Yaxian Wu, Yi Xiao, Jun An

**Affiliations:** ^1^ Department of Cardiothoracic Surgery, The Third Affiliated Hospital of Sun Yat-sen University, Guangzhou, China; ^2^ Department of Clinical Laboratory, Sun Yat-sen University Cancer Center, State Key Laboratory of Oncology in South China, Collaborative Innovation Center for Cancer Medicine, Guangzhou, China; ^3^ Department of Microbial and Biochemical Pharmacy, School of Pharmaceutical Sciences, Sun Yat-sen University, Guangzhou, China

**Keywords:** esophageal squamous cell carcinoma, *METTL3*, m6A modification, immune infiltration, immunotyping, prognostic model

## Abstract

Methyltransferase-like 3 (*METTL3*) is an RNA methyltransferase mediating N6 methyladenosine (m6A) modification. Its role in cancer pathogenesis and progression has attracted increasing attention. However, the immunological role, possible immune mechanism, and clinical significance of *METTL3* in esophageal squamous cell carcinoma (ESCC) remain to be confirmed. The Tumor Genome Atlas (TCGA) provided clinical and transcriptome sequencing data for this study (162 tumor tissue samples and 11 normal tissue samples), while the Immunology Database and Analysis Portal (immport, https://www.immport.org/home) provided 2483 immune-related genes. *METTL3* was substantially expressed in ESCC and linked to poor prognosis in ESCC, according to the findings. Functional analysis revealed that *METTL3* is mainly involved in chromosomal homologous recombination and DNA mismatch repair processes, which could be potential mechanisms for tumor disease development and progression. Analysis on the TISIDB website shows that effector memory CD8 T cells, NK cells, neutrophils and other cells are highly correlated with *METTL3* expression. We screened immune genes associated with *METTL3* by Spearman’s analysis and performed functional analysis. These immune genes were mostly linked with immune processes, such as cytokine receptors, the *MAPK* signaling pathway, and natural killer cell-mediated cytotoxicity, indicating that *METTL3* is a key molecule in the immune regulation of esophageal cancer. In addition, based on *METTL3*-related immune genes, we separated the patients into several subgroups and constructed a prognostic prediction model consisting of six immune genes. As an independent prognostic indicator for ESCC, the risk score of this model can be employed. A nomogram was also developed to accurately evaluate individual prognoses based on clinical indicators and risk scores. In summary, this study suggests that *METTL3* is not only a potential pathogenic molecule for esophageal carcinogenesis and progression but also a potential biological marker for forecasting ESCC patient prognosis and could serve as a basis for clinical decision making.

## Introduction

Esophageal carcinoma (EC) is among the most prevalent upper gastrointestinal tract malignancies worldwide. It is the eighth most frequent cancer worldwide, as well as the sixth leading cause of cancer-related mortality, and the incidence rate is still rising (1–3). In East Asia, especially in China, the incidence rate of EC is very high, and one-third to one-half of EC cases around the world originate in China (4). The most common histological type of EC in China is esophageal squamous cell carcinoma (ESCC), which accounts for 90% of all EC cases (5, 6).

Epigenetics is a field of biology that has rapidly developed in recent years. Its roles in tumor formation and progression have been increasingly recognized (7–9). Epigenetic regulation mechanisms mainly include RNA modification, DNA modification, histone posttranslational modifications, and chromatin remodeling. Epigenetic regulation of gene expression is a dynamic and reversible process (10–12). In biological and pathological processes, the related modifications are crucial for interpreting environmental signals and altering the expression of related genes (13, 14). When these genes are dysregulated, diseases such as ESCC can occur (15–19).

Of more than 170 known RNA modifications, the most widespread mRNA modification, N6-methyladenosine (m6A), has an impact on practically every stage of RNA metabolism involving splicing, decay, export, and translation (20, 21). M6A is dynamically installed and deleted by writer and eraser enzymes, and it acts by recognizing and binding to m6A reader proteins. Methyltransferase-like 3 (*METTL3*) is the main methyltransferase critical for m6A methylation (22, 23). *METTL3* dysregulation has already been widely reported in a wide range of tumor types, such as lung cancer, colorectal cancer and breast cancer (24–28). However, there are still few studies of the immune function and mechanism of *METTL3* in ESCC.

In this research, the RNA sequencing data and the clinical information of ESCC patients were acquired from the Cancer Genome Atlas (TCGA). *METTL3* gene expression and function (especially immune function) were analyzed. The screened immune-related prognostic genes were also used in consensus clustering analysis and least absolute shrinkage and selection operator (LASSO) regression analysis to build a tumor immune subtype and risk score model to predict ESCC patient prognosis.

## Materials and Methods

### Acquisition of Data and Tissue Specimens

The TCGA database provides ESCC patient gene expression patterns and clinical data (https://cancergenome.nih.gov/) (29). The dataset included information about 162 tumor tissue samples and 11 normal tissue samples. Additionally, from the Immunology Database and Analysis Portal (IMMPORT, https://www.immport.org/home), 2483 immune-related genes were imported and used for further immunological research (30).

At the Third Affiliated Hospital of Sun Yat-sen University from 2019 to 2021, four pairs of tumor and adjacent tissues were collected. The stage of each sample was confirmed by pathological examination, as stated by the American Joint Commission on Cancer (AJCC) cancer staging manual (8th Edition). For biological research, written informed consent was acquired from the patient or guardian. All of the experiments were authorized by the ethical committee of Sun Yat-sen University’s Third Affiliated Hospital.

### Bioinformatic Analysis

Wilcoxon’s signed-rank test was applied for the differential expression analysis of *METTL3* and immune cells (using the limma package). The prognostic value of *METTL3*, the risk score model and the nomogram were expounded by survival analysis (Kaplan-Meier method and log-rank test, using the survival and survminer packages) and receiver operating characteristic (ROC) curve analysis (using the time ROC package). Analysis of whether *METTL3* and *METTL3*-related immune genes were associated with patient prognosis was conducted using Cox regression (univariate and multivariate, using the survival package). The role of *METTL3* in ESCC was analyzed using gene set enrichment analysis (GSEA, http://www.gsea-msigdb.org/gsea/index.jsp) (31, 32).

The infiltration level of immune cells was calculated using the CIBERSORT RNA deconvolution algorithm (using the e1071 and preprocessCore packages) (33, 34). Using Pearson’s test, we analyzed the correlation of different immune cells and screened *METTL3-*related immune genes. In addition, the TISIDB database (http://cis.hku.hk/TISIDB) was utilized to screen immune cells closely linked to *METTL3* (35). The biological pathways of *METTL3*-related genes were further assessed by Gene Ontology (GO) and Kyoto Encyclopedia of Genes and Genomes (KEGG) gene enrichment analyses (using the enrichplot and clusterProfiler packages, respectively) (36–39). The Search Tool for the Retrieval of Interacting Genes/Proteins database (STRING, https://cn.string-db.org/) was used to study gene interactions (40, 41)

The patients with ESCC were divided into different subgroups by consensus clustering analysis (using the ConsensusClusterPlus package) (42, 43). Principal component analysis (PCA) was used to verify the significance of typing (using scatterplot3d packages) (44, 45). The risk score model was created by least absolute shrinkage and selection operator (LASSO) Cox regression analysis (using the glmnet package) (46). A nomogram was generated by combining the risk score with various clinical data (using the RMS package) (47, 48).

### Cell Culture

The American Type Culture Collection (ATCC) provided Eca109 cells. Cells were grown in RPMI 1640 medium (Invitrogen, USA). All of the media were mixed with 10% fetal bovine serum (FBS, Gibco, USA), and all of the cells were incubated with 5% CO_2_ at 37°C.

### Establishment of Stable Cell Lines

pcDNA/*METTL3* or a vector control was used to establish stable cell lines. Stable Eca109 cell lines that overexpressed *METTL3* were generated by lentiviral transduction in the presence of 1 μg/mL polybrene (Sigma, USA). *METTL3* stable knockdown involves short hairpin RNA expressed from a lentiviral vector. Lentiviral particles generated by the transfection of the second-generation *sh-METTL3* transfer vector plasmid, packaging plasmid psPAX2 and envelope plasmid pMDG.2 (3:2:1) into a lentiviral vector-producing cell line were used to transduce Eca109 cells. Empty vector was used as a control. The cells were transduced in the presence of 1 μg/mL polybrene (Sigma, USA), selected with puromycin, and expanded to produce a stable cell line. The target sequence of the shRNA was as follows: *shMETTL3*: GCCAAGGAACAATCCATTGTT.

### Western Blotting Analysis

Cellular proteins were separated using 10% SDS–PAGE, transferred to polyvinylidene difluoride membranes and probed with antibodies against *METTL3* (1:2,000, CST). Anti-rabbit antibodies coupled with horseradish peroxidase (HRP) (1:1,000, BOSTER) were used as secondary antibodies. Specific antibodies were used to measure GAPDH protein levels (1:5,000, CST).

### Wound Healing Assay

To generate a confluent monolayer, cells were plated in 60-mm dishes. Then, a 10μl pipette tip was used to scratch the cell monolayer in a straight line. The plate was washed gently twice before being incubated at 37°C with 5% CO_2_ in 1640 supplemented with 1% FBS. The outcomes were observed at 0 and 48 hours with a microscope. Each test was performed three times.

### Cell Migration Assay

Transwell chambers (Corning, USA) were utilized to test cell migration. In the upper chamber, Eca109 cells (1 × 10^5^) were cultured in serum-free media, while in the lower chamber, 20% FBS was added to RPMI 1640 medium. Invasive cells in the lower chambers were fixed with 4% paraformaldehyde and stained with 0.05% crystal violet (Sigma-Aldrich) after being incubated for 24 hours at 37°C. Five random fields of cells were counted under an inverted microscope (Leica DMI4000B, Germany).

### Histological Immunohistochemical Assays

At 4°C overnight, tissue slices were treated with primary antibodies against *METTL3* (1:50, CST). After washing with PBST, an HRP-conjugated anti-rabbit secondary antibody (1:5,000, BOSTER) was applied to the sections at room temperature for 1 h. Before counterstaining with 10% Mayer’s hematoxylin, the sections were reacted with 3-diaminobenzidine tetrahydrochloride for 10 s. Two experienced pathologists analyzed the IHC results. To quantify the percentage of positively stained cells versus the total number of tumor cells, visual fields (×400 magnification) were selected.

### Statistical Analysis

For data analysis and statistics, R software (version 3.6.3; the R Foundation for Statistical Computing) and SPSS software (version 23.0; IBM Corp, New York, USA) were used. Student’s t test or Wilcoxon’s signed-rank test was utilized to perform statistical comparisons between groups. The Kaplan-Meier method and log-rank test were used for survival analysis. Receiver operating characteristic (ROC) curve analysis was used to check performance. Prognostic variables were screened using Cox regression (univariate and multivariate) analysis. For correlation analysis, Pearson’s test was utilized. The CIBERSORTR deconvolution algorithm was utilized to calculate the degree of immune cell infiltration. Consensus clustering analysis was utilized to create subtypes. The risk score model was created using LASSO regression analysis. A *P* value less than 0.05 was considered significant.

## Results

### *METTL3* Is Overexpressed in ESCC

**Figure 1:**
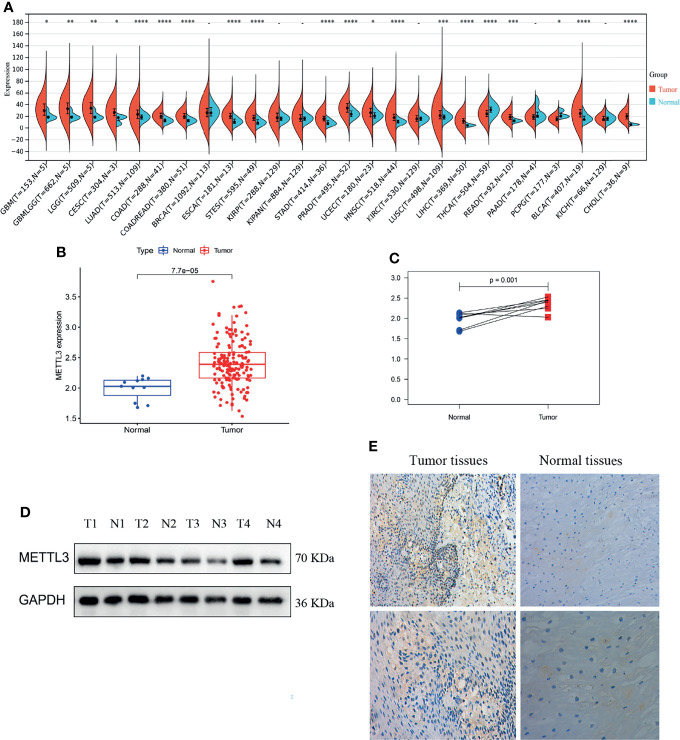
*METTL3* was overexpressed in tumor tissues. **(A–C)** Differential expression of *METTL3* between tumor tissues and normal tissues (the normal tissue is represented by blue, whereas the tumor tissue is shown in red; *P < 0.05, **P < 0.01, ***P < 0.001 and ****P < 0.0001. -, not significant). **(D, E)** Immunohistochemical staining and western blotting of ESCC tissues (n = 4) and adjacent tissues (n = 4).

First, we analyzed the differential expression of *METTL3* across cancer datasets. *METTL3* expression was considerably higher in liver cancer, cholangiocarcinoma, colon cancer, and other tumor tissues than in normal tissues (*P < 0.05, **P < 0.01, ***P < 0.001 and ****P < 0.0001; -, not significant, **Figure 1A**). Then, ESCC *METTL3* expression was analyzed. The results revealed that ESCC tissues showed a high level of *METTL3* expression (*P* = 7.7e-05, **Figure 1B**). The same result was found in the paired samples (*P* = 0.001, **Figure 1C**). Immunohistochemical staining and western blotting were utilized to examine *METTL3* expression in ESCC tissues (n = 4) and normal tissues (n = 4). *METTL3* expression was particularly high in tumor tissues (**Figures 1D, E**).

### High *METTL3* Expression Can Promote ESCC Progression

**Figure 2:**
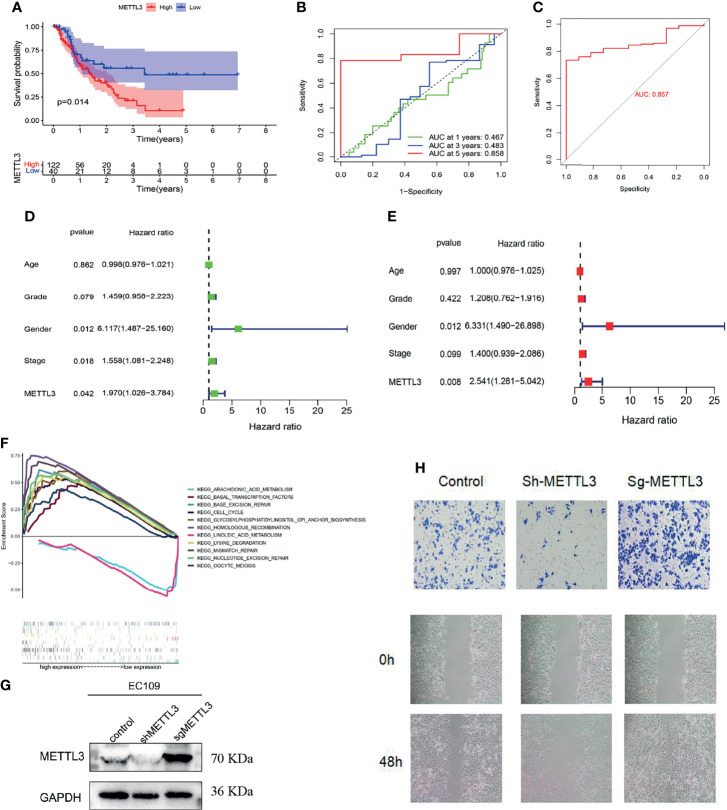
Function and clinical significance of *METTL3* in ESCC. **(A)** PFS analysis of *METTL3* in the high and low expression groups (*P* = 0.014). **(B, C)** ROC curve analysis to evaluate the prognostic performance (AUC = 0.858) and diagnostic performance (AUC = 0.857) of *METTL3* in ESCC. **(D, E)** Cox regression (univariate and multivariate) analysis indicated that *METTL3* could be utilized as a prognostic marker for ESCC (using PFS data, *P* = 0.008, HR = 2.541). **(F)** Analysis of *METTL3* functionality through GSEA. **(G, H)** *METTL3*’s migration ability was identified using wound healing and cell migration assays.

Based on the median value of *METTL3* expression, ESCC patients were divided into two groups: high expression and low expression. After analyzing the PFS (progression-free survival) of patients, the Kaplan-Meier algorithm was adopted. The findings revealed that the PFS of *METTL3*-overexpressing patients was markedly lower than that of low *METTL3*-expressing patients (*P* = 0.014, **Figure 2A**), indicating that *METTL3*-overexpressing patients have a poorer prognosis.

According to ROC curve analysis, *METTL3* not only had high accuracy in predicting the 5-year survival of patients with ESCC (area under the curve AUC = 0.858, **Figure 2B**) but also had important guiding significance for the diagnosis (AUC = 0.857, **Figure 2C**). Cox regression (univariate and multivariate) analysis also indicated that *METTL3* could be utilized as a prognostic marker for ESCC (using PFS data, *P* = 0.008, HR = 2.541, **Figures 2D, E**). Furthermore, the gene function differences between the high and low *METTL3* expression groups were examined by Gene Set Enrichment Analysis (GSEA). We discovered that *METTL3* is primarily implicated in chromosomal homologous recombination and DNA mismatch repair, which could be potential mechanisms for the occurrence and progression of tumor diseases (**Figure 2F**). The wound healing assay and cell migration assay of Eca109 cells also showed that *METTL3* overexpression could promote the migration of ESCC cells, while low *METTL3* expression inhibited the occurrence of these conditions (**Figures 2G, H**).

### ESCC Can Be Infiltrated by a Variety of Immune Cells, Some of Which Are *METTL3*-Related

**Figure 3:**
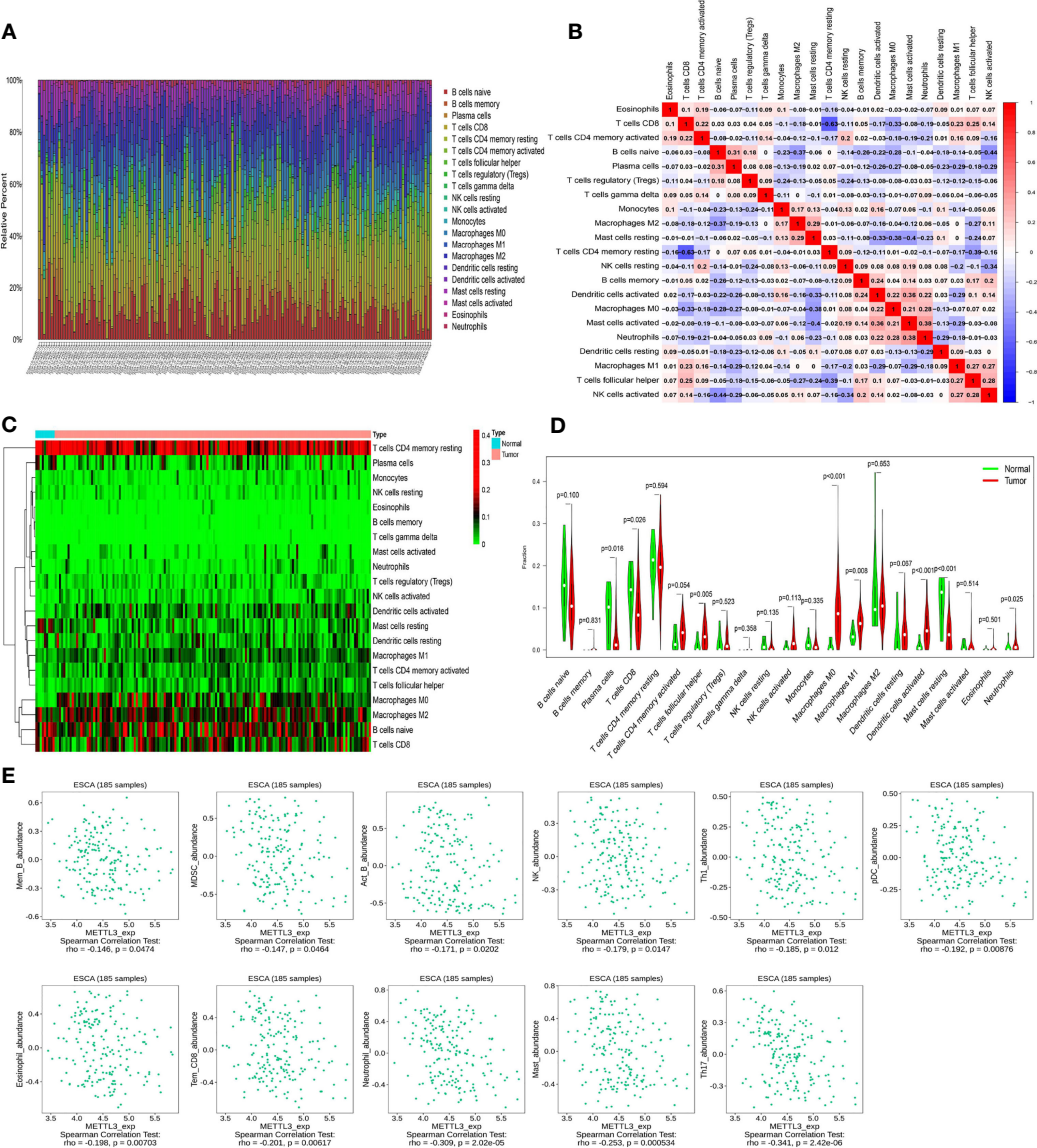
Analysis of immune cell infiltration and *METTL3*-related immune cells in ESCC. **(A)** The degree of immune cell infiltration in esophageal cancer (different colors represent different immune cells). **(B)** Correlation analysis between different immune cells (red and blue represent positive and negative correlations, respectively, and correlation coefficients are expressed numerically). **(C, D)** The differential enrichment of immune cells in ESCC and normal tissues. **(E)** Correlation between *METTL3* and immune cells.

The infiltration levels of 21 immune cells in tumors were calculated by the CIBERSORT method. The analysis revealed a high degree of macrophage, T cell, and B cell infiltration (**Figure 3A**). Subsequently, the correlation analysis revealed that immune cells had varying degrees of correlation (**Figure 3B**). Comparing immune cell levels in the tumor and normal groups, the results showed that plasma cells (*P* = 0.016), CD8 T cells (*P* = 0.026), follicular helper T cells (*P* = 0.005), M0 macrophages (*P* < 0.001), M1 macrophages (*p* = 0.008), activated dendritic cells (*P* < 0.001), resting mast cells (*P* < 0.001), and neutrophils (*P* = 0.025) were significantly differentially enriched (**Figures 3C, D**).

In addition, immune cells closely linked to *METTL3* were screened using the TISIDB database (http://cis.hku.hk/TISIDB). The results revealed that effector memory CD8 T cells (*P* = 0.00617), NK cells (*P* = 0.0147), neutrophils (*P* = 2.02e-05) and other cells were highly correlated with *METTL3* expression (**Figure 3E**), indicating that the *METTL3* gene could have a crucial impact on the cellular immune regulation process of ESCC.

### *METTL3* Has a Critical Function in ESCC Immune Modulation

**Figure 4:**
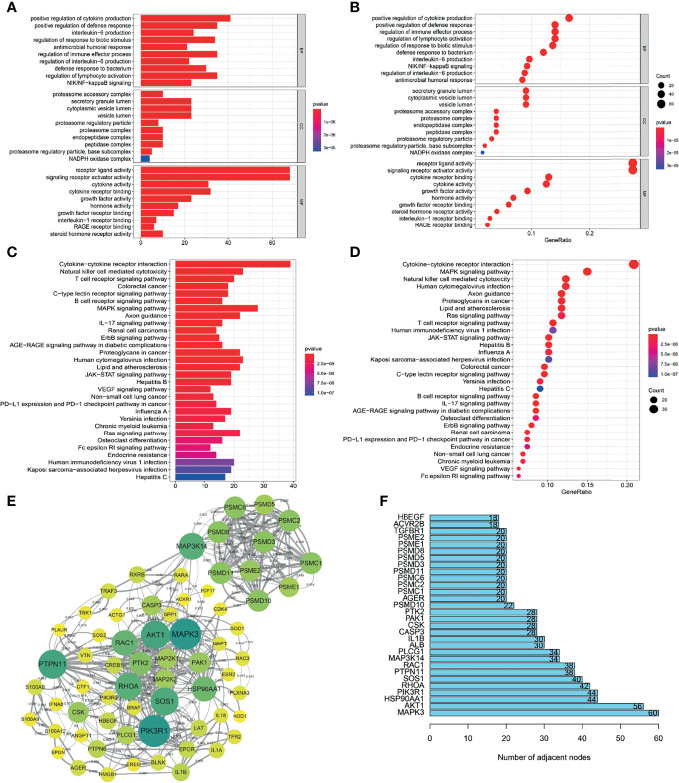
Screening and functional analysis of immune genes highly related to *METTL3.* **(A, B)** GO analysis of *METTL3*-related immune genes. **(C, D)** KEGG analysis of *METTL3*-related immune genes. **(E)** Protein-protein interaction network analysis of *METTL3*-related immune genes (high confidence 0.700; only some core genes were displayed; the larger the circle, the darker the color, representing the more adjacent nodes). **(F)** The core genes of the protein-protein interaction network (showing only the top 30).

To understand how the *METTL3* gene regulates the immune system in ESCC, we analyzed the correlations between the *METTL3* gene and immune genes in the ImmPort database. As a result, 261 highly related immune genes were screened (*P* < 0.05) (**Supplementary Table 1**). The Gene Ontology terms (GO terms) of 261 immune genes demonstrated that these genes were mostly expressed in the cytoplasm, related to receptor ligand activity and signal receptor activator activity, and they participated in biological processes such as cytokine secretion, lymphocyte activation and immune effect process regulation (**Figures 4A, B**). Kyoto Encyclopedia of Genes and Genomes (KEGG) analysis showed that immunomodulatory processes, such as cytokine–cytokine receptor interaction, the *MAPK* signaling pathway and natural killer cell-mediated cytotoxicity, were also related to these genes (**Figures 4C, D**).

The STRING website (https://cn.string-db.org/) was applied to examine the protein interaction relationships of these immune genes. We obtained a protein interaction network diagram (**Figure 4E**). In addition, the number of connection nodes of each protein was counted to clarify the core protein of the protein interaction network. The results showed that *MAPK3, AKT1, HSP90AA1, PIK3R1* and *RHOA* were the most important core genes (**Figure 4F**). According to the above analysis, *METTL3* has a significant impact on the immune regulation of ESCC.

### ESCC Can Be Classified Into Three Immune Subtypes With Different Prognoses

**Figure 5:**
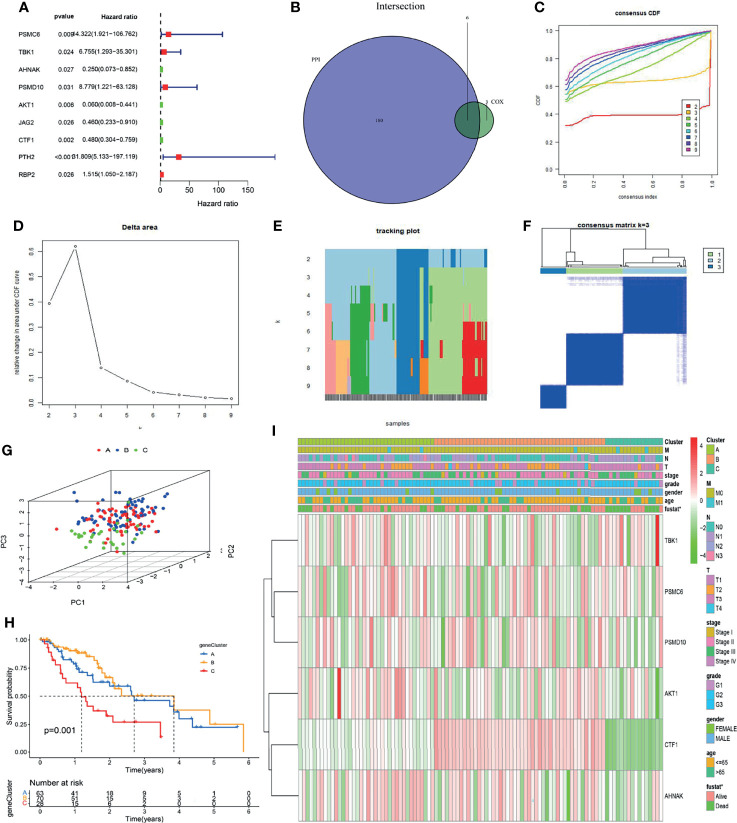
Six immune genes were screened and tumors were classified into three immune subtypes. **(A)** Using univariate Cox analysis, 9 immunological genes associated with prognosis were identified. **(B)** The intersection between 180 PPI network core genes and 9 immune genes was selected, and 6 immune genes were screened. **(C–F)** Based on 6 immune genes, ESCC could be classified into three immune subtypes by consensus clustering analysis. **(G)** Principal component analysis (PCA) showed that the tumors were well typed. **(H)** OS analysis of patients with different subtypes (*P* = 0.001). **(I)** Gene expression analysis and clinical correlation analysis of different subtypes (*P < 0.05).

We explored the clinical significance of immune genes highly related to *METTL3* by Cox univariate analysis and identified 9 immune genes related to prognosis (*PSMC6*, *TBK1*, *AHNAK*, *PSMD10*, *AKT1*, *JAG2*, *CTF1*, *PTH2*, *RBP2*) (**Figure 5A**). Then, these genes overlapped with the core genes of the protein interaction network, and 6 genes (*PSMC6*, *TBK1*, *AHNAK*, *PSMD10*, *AKT1* and *CTF1*) were finally obtained (**Figure 5B** and **Supplementary Table 2**). Using the ConsensusClusterPlus package and K-means (KM) algorithm, consensus clustering analysis according to the 6 genes was performed. As a result of the expression of the 6 genes, the samples were divided into three distinct subtypes: A, B, and C (**Figures 5C–F**). Principal component analysis (PCA) showed significantly distinct subtypes (**Figure 5G**). Overall survival (OS) analysis of the three subtypes showed that patients with types A and B had a better prognosis than those with type C (*P* = 0.001, **Figure 5H**). In addition, the thermogram of tumor subtypes showed a correlation between patient survival status and subtype (*P* < 0.05, **Figure 5I**). The above analysis showed that tumor typing according to 6 immune genes has guiding significance for patient prognosis evaluation.

### The Risk Score Model Was Used to Identify ESCC Patients With Poor Prognosis

**Figure 6:**
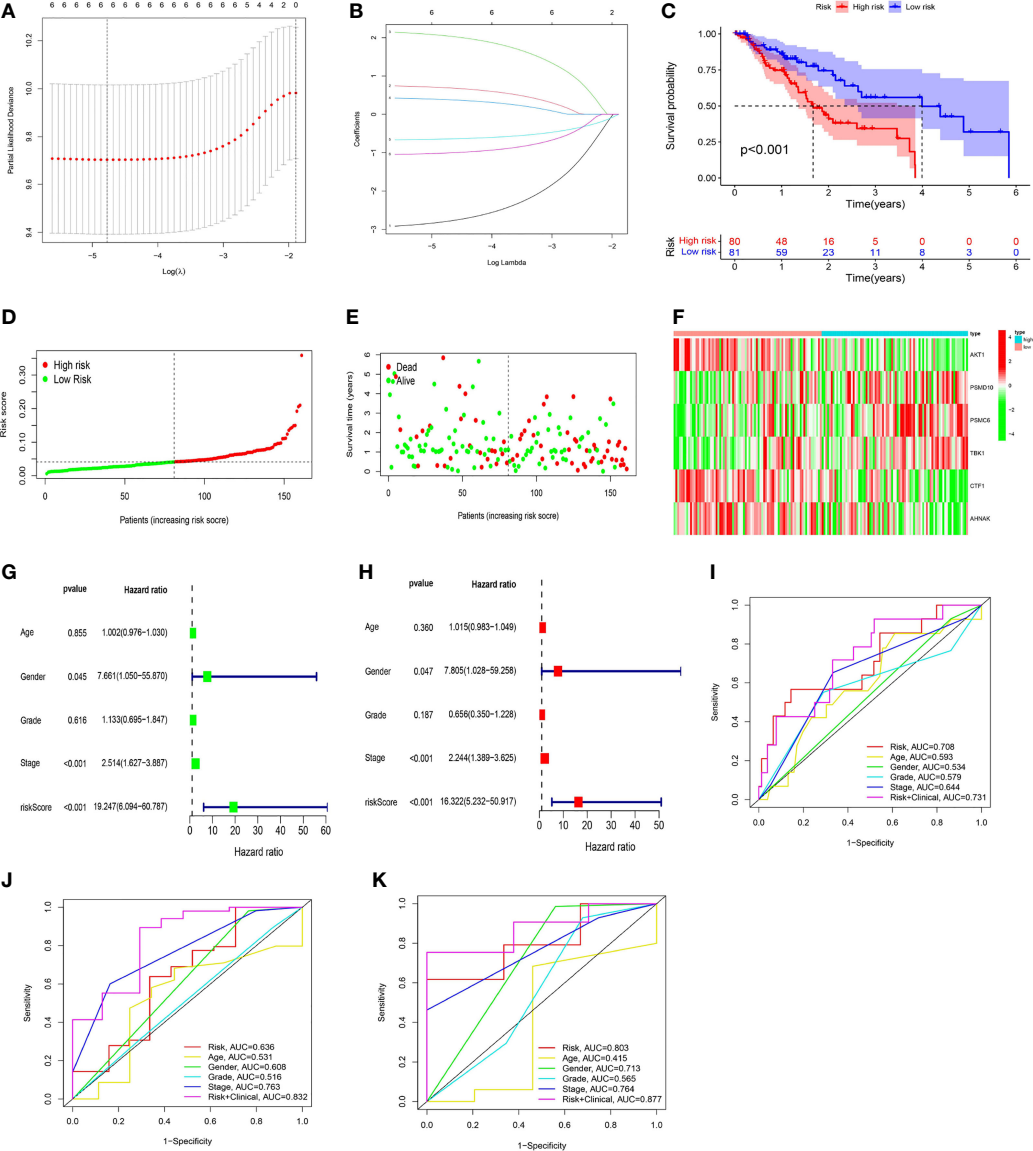
The development of a risk scoring model. **(A, B)** LASSO regression analysis was used to create a risk scoring model that worked best when all 6 immune genes were included in the model. **(C)** The low-risk group had a higher overall survival rate than the high-risk group (*P* < 0.001). **(D, E)** On the risk curve, a higher risk score indicated a shorter survival time and a higher mortality rate. **(F)** In the high-risk group, the expression levels of *PSMD10*, *PSMC6*, and *TBK1* were upregulated, and the expression levels of *AKT1*, *CTF1*, and *AHNAK* were downregulated. **(G, H)** Cox regression (univariate and multivariate) analysis indicated that gender (*P* = 0.047, HR = 7.805), stage (*P* < 0.001, HR = 2.244), and risk score (*P* < 0.001, HR = 16.322) could be utilized as independent prognostic markers for ESCC patients (using OS data). **(I–K)** The risk score’s accuracy in predicting prognosis was assessed using ROC curves (I: 1-year; J: 3-year; K: 5-year).

Based on the above 6 immune genes related to prognosis, LASSO regression analysis was used to create a risk scoring model that worked best when all 6 immune genes were included in the model. The risk coefficient of each immune-related prognostic gene was calculated, and the risk scoring equation was obtained: risk score = 2.05 * *PSMC6* + 0.69 * *PSMD10* + 0.38 * *TBK1*-0.65 * *CTF1*-1.01 * *AHNAK*-2.79 * *AKT1*. The risk scoring model was established (**Figures 6A, B**).

The median risk score was used to categorize ESCC patients into high-risk and low-risk groups. Regarding the overall survival (OS) curve, the low-risk group had a higher survival rate (*P* < 0.001, **Figure 6C**). Regarding the risk curve, a higher risk score indicated a shorter survival time and a higher mortality rate (**Figures 6D, E**). In addition, *PSMC6*, *PSMD10*, and *TBK1* were highly expressed in the high-risk group, while *CTF1*, *AHNAK*, and *AKT1* were highly expressed in the low-risk group, according to the gene expression heatmap (**Figure 6F**).

Cox regression (univariate and multivariate) analysis also indicated that gender (*P* = 0.047, HR = 7.805), stage (*P* < 0.001, HR = 2.244), and risk score (*P* < 0.001, HR = 16.322) could be utilized as prognostic markers for ESCC patients (using OS data, **Figures 6G, H**). ROC curve analysis showed that, when the risk score was combined with age, gender, grade, stage and other indicators to comprehensively evaluate the prognosis of EC patients, it had high accuracy. The 1-year ROC curve had an AUC of 0.731 (**Figure 6I**), the 3-year ROC curve had an AUC of 0.832 (**Figure 6J**), and the 5-year ROC curve had an AUC of 0.877 (**Figure 6K**), showing a gradual improvement in accuracy.

### A Nomogram Can Reliably Predict the Prognosis of ESCC Patients

**Figure 7:**
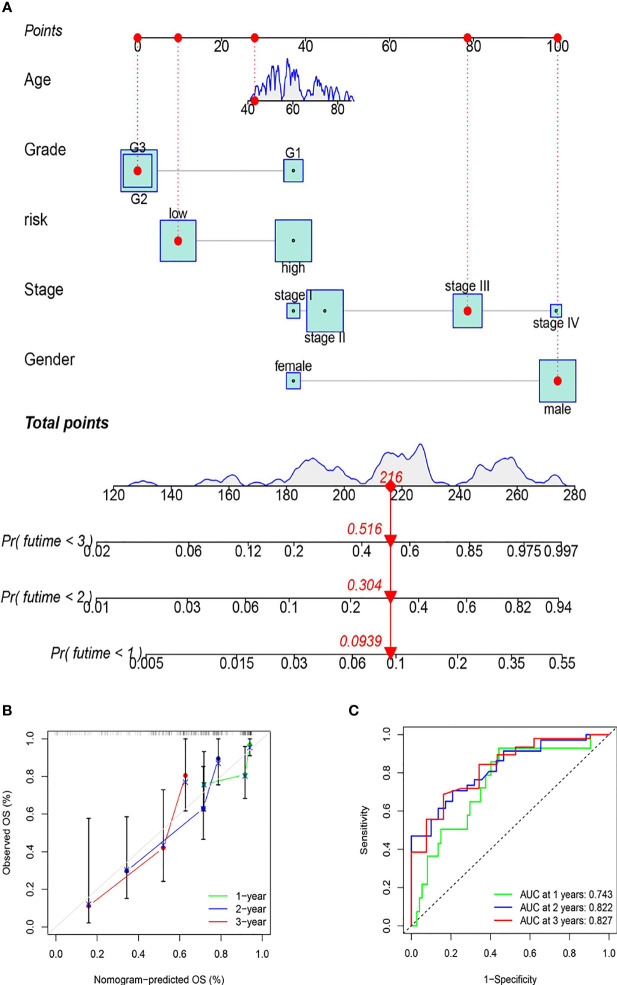
The establishment of the nomogram. **(A)** Prognostic prediction mechanism of the nomogram (the various elements were scored and summed to produce a total score, and patient survival was predicted based on the total score). **(B)** The nomogram’s accuracy in predicting prognosis was validated by calibration curves. **(C)** The nomogram’s accuracy in predicting prognosis was assessed using ROC curve analysis.

To more accurately assess the prognosis of patients with ESCC, the risk score and clinical characteristics were used to create a nomogram. The various elements were scored and summed to produce a total score, and patient survival was predicted based on the total score (**Figure 7A**). In addition, the nomogram’s application ability was validated by calibration curves and ROC curves.

As shown by the calibration curves, the prognosis prediction of the nomogram for patients at 1, 2, and 3 years is close to the observation, showing that the model has high accuracy (**Figure 7B**). As shown by the ROC curves, the 1-year ROC curve had an AUC of 0.743, the 2-year ROC curve had an AUC of 0.822, and the 3-year ROC curve had an AUC of 0.827, proving the high accuracy of the nomogram (**Figure 7C**). The results of this study revealed that the nomogram can reliably predict the prognosis of ESCC patients.

## Discussion

ESCC is an invasive disease with high malignancy potential and a dismal prognosis. The treatment of esophageal cancer mainly includes surgery, radiotherapy, chemotherapy, targeted therapy and so on. Despite recent advances in the treatment of esophageal cancer, the prognosis for esophageal cancer remains relatively poor (49, 50). In recent years, immunotherapy for EC has attracted increasing attention, and various clinical studies have been performed (51, 52). However, immunotherapy for EC always leads to mixed results, partly due to the lack of reliable markers for predicting treatment response (53). In the last several years, an increasing number of studies have been devoted to identifying differential biomarker candidates, especially immune-related genes (54).

Methyltransferase-like 3 (*METTL3*) is an RNA methyltransferase that mediates N6 methyladenosine (m6A) modification. Its role in cancer pathogenesis and progression has attracted increasing attention (24, 55). However, the immunological role, possible immune mechanism, and clinical significance of *METTL3* in esophageal squamous cell carcinoma (ESCC) remain to be confirmed.

Therefore, it is necessary to analyze this topic and understand the immune function and molecular mechanism of *METTL3* in esophageal cancer to explore whether *METTL3* could become a new biomarker for immunotherapy of esophageal cancer and provide a new basis for immunotherapy and individualized treatment of ESCC patients.

In this investigation, *METTL3* expression was shown to be much higher in cancerous tissue than in healthy tissue. *METTL3* overexpression predicted a poor prognosis and is an independent prognostic factor. GSEA revealed that *METTL3* is mainly involved in chromosome homologous recombination and DNA mismatch repair, which could be potential mechanisms for the occurrence and progression of tumor-related diseases.

Based on CIBERSORT and TISIDB analysis, T lymphocytes, B lymphocytes, and neutrophils had high levels of infiltration in ESCC, and their infiltration was demonstrated to be negatively related to *METTL3* expression. *METTL3* was linked with immune processes, such as cytokine receptor interaction, the *MAPK* signaling pathway, and natural killer cell-mediated cytotoxicity, as determined by GO/KEGG functional enrichment analysis.

*METTL3*-related immune prognostic genes were used to cluster ESCC patients. Survival rates for patients with ESCC in subgroup A and B were considerably greater than for those in categories C, making this finding particularly instructive in such patients’ evaluation and treatment. In addition, we constructed a prognosis prediction model composed of six genes (*PSMC6*, *TBK1*, *AHNAK*, *PSMD10*, *AKT1* and *CTF1*). Based on the results of univariate and multivariate Cox analyses, the risk score calculated according to the model equation is a good independent survival index. Forecasting patient survival and assessing probable clinical outcomes are both possible uses for the risk score tool. OS analysis and ROC curve analysis also verified that the model had good prediction performance. In addition, the risk score combined with clinical indicators constructed into a nomogram also had superior prognostic predictive power, as verified by the calibration curve and ROC curve analysis.

Among the genes involved in the construction of the prognosis prediction model, *PSMC6* encodes the proteasome 26S subunit and is involved in the ATP-dependent degradation of ubiquitinated proteins. Thus, the proteasome participates in cell cycle progression, apoptosis, and DNA damage repair. Studies have shown that *PSMC6* is associated with lung adenocarcinoma, breast cancer, pheochromocytoma, low-grade glioma, colorectal melanoma and other diseases (56–59).

TANK-binding kinase 1 (*TBK1*) is a Ser/Thr kinase with a central role in coordinating the cellular response to invading pathogens and regulating key inflammatory signaling cascades. Some genes play an important role in the antiviral mechanism of cells, such as *TBK1*, *IRGM* and so on (60, 61). Studies have shown that *TBK1* is also associated with cancers, such as kidney cancer, cervical cancer, and lung cancer (62–64).

*AHNAK* encodes a large (700 kDa) structural scaffold protein. The protein might play a role in blood-brain barrier formation, cell structure and migration, cardiac calcium channel regulation, and tumor metastasis. Studies have shown that *AHNAK* is associated with colorectal cancer, ovarian cancer, gastric cancer and other diseases (65–68).

*PSMD10* encodes a subunit of the PA700/19S complex, which is a regulatory component of the 26s proteasome and could be involved in protein-protein interactions. Aberrant expression of this gene could play a role in tumorigenesis. Studies have shown that *PSMD10* is associated with tumor diseases, such as hepatocellular carcinoma and thyroid cancer (69–72).

Akt serine/threonine kinase 1 (*AKT1*) encodes a member of the human Akt serine threonine protein kinase family. It can be phosphorylated by phosphoinositide 3 kinase (PI3K) and participate in the AKT/PI3K pathway. It is a key part of many signal transduction pathways and is associated with diseases such as gastric, prostate, breast and ovarian cancers (73–75).

The *CTF1* gene encodes a secretory cytokine capable of inducing cardiomyocyte hypertrophy *in vitro*, and it plays roles in hypertensive heart disease, dilated cardiomyopathy, lung adenocarcinoma and other diseases (76–78).

It is worth mentioning that, while the primary functions of these genes are distinct, we reviewed previous reports that these six genes are associated with cancer patient prognosis. Therefore, the prognostic prediction model relying on these six genes has a high degree of accuracy and reliability.

However, our research still has some shortcomings. First, although we included all esophageal cancer cases (containing 162 tumor tissue samples and 11 normal tissue samples) from the Tumor Genome Atlas (TCGA) database in the study, the number of cases still must be further increased, and we will include Gene Expression Omnibus (GEO), International Cancer Genome Consortium (ICGC) and other databases for further research. Second, although we have performed some experiments to verify the analysis results, more experiments are needed to strengthen the credibility, which will be the focus of our next work, and some experiments have already been performed.

## Conclusion

In summary, this study suggests that *METTL3* is not only a potential pathogenic molecule for esophageal carcinogenesis and progression but also a potential target for immunotherapy in esophageal cancer. In addition, *METTL3* is also a biomarker for forecasting ESCC patient prognosis. The established subtype delineation system and prognostic prediction model can be utilized to predict the prognosis of patients and assess the potential clinical risk. These findings could help to provide a new basis for immunotherapy and individualized treatment of ESCC patients.

## Data Availability Statement

The datasets presented in this study can be found in online repositories. The names of the repository/repositories and accession number(s) can be found in the article/**Supplementary Material**.

## Ethics Statement

The studies involving human participants were reviewed and approved by The Third Affiliated Hospital, Sun Yat-sen University. The patients/participants provided their written informed consent to participate in this study

## Author Contributions

YZ was a major contributor to all of the experimental work, data analysis, and manuscript writing. SG and YL were involved in the experimental work. FC, YW and YX were involved in the data analysis. JA conceptualized the project, acquired funding, and assisted with the manuscript development. The final manuscript was reviewed and approved by all of the authors.

## Funding

The current study was funded by the Natural Science Foundation of Guangdong Province, China (No. 2017A030313118).

## Conflict of Interest

The authors declare that the research was conducted in the absence of any commercial or financial relationships that could be construed as a potential conflict of interest.

## Publisher’s Note

All claims expressed in this article are solely those of the authors and do not necessarily represent those of their affiliated organizations, or those of the publisher, the editors and the reviewers. Any product that may be evaluated in this article, or claim that may be made by its manufacturer, is not guaranteed or endorsed by the publisher.

## Abbreviations

EC, esophageal carcinoma; ESCC, esophageal squamous cell carcinoma; *METTL3*, methyltransferase-like 3; M6A, n6 methyladenosine; TCGA, the Tumor Genome Atlas; IMMPORT, the immunology database and analysis portal; OS, overall survival; PFS, progression-free survival; ROC, receiver operating characteristic; AUC, area under curve; GO, gene ontology; KEGG, Kyoto Encyclopedia of Genes and Genomes; GSEA, gene set enrichment analysis; PCA, principal component analysis; LASSO, the least absolute shrinkage and selection operator; HR, hazard ratio.
